# Asian koel rapidly locates host breeding in novel nest sites

**DOI:** 10.1002/ece3.11345

**Published:** 2024-04-29

**Authors:** Mominul Islam Nahid, Peter S. Ranke, Wei Liang

**Affiliations:** ^1^ Ministry of Education Key Laboratory for Ecology of Tropical Islands, Key Laboratory of Tropical Animal and Plant Ecology of Hainan Province, College of Life Sciences Hainan Normal University Haikou China; ^2^ Bangladesh Forest Research Institute (BFRI) Khulna Bangladesh; ^3^ Centre for Biodiversity Dynamics (CBD), Department of Biology Norwegian University of Science and Technology (NTNU) Trondheim Norway; ^4^ BirdLife Norway Trondheim Norway

**Keywords:** Asian koel, common myna, host activity, nest box, nest type

## Abstract

Avian brood parasites depend upon locating host nests to lay their eggs. However, how brood parasites locate host nests and select the nests for parasitism remains poorly studied. Here, we examined how a non‐evicting brood parasite, the Asian koel (*Eudynamys scolopaceus*) were able to locate host nests in locally novel nest sites. We provided a set of novel nest sites (i.e., nestboxes, *n* = 100) to the common myna (*Acridotheres tristis*), a regular host of the Asian koel, at a field site in Central Bangladesh. We found that common myna quickly utilized the novel breeding sites (*n* = 99 nests across 59 boxes) and similarly, 21.2% of these nests were parasitized by the Asian koel. Unsurprisingly, none of the inactive nest boxes were parasitized, neither were empty nests of common myna (i.e., either predated or fledged clutches, thus lack of parental activity, *n* = 83). Our results provide an experimental demonstration of the Asian koel ability to rapidly locate nests in locally novel nest sites, potentially facilitated by close monitoring of host activity and behavior around the nest site. However, the underlying mechanisms for this rapid adjustment to novel nest sites remain unresolved.

## Graphical abstract

Common mynas (*Acridotheres tristis*) built nests in the boxes and 21.2% of these were parasitized by the Asian koel (*Eudynamys scolopaceus*), while none of non‐host species nests were parasitized. Active boxes were significantly more parasitized than inactive nest boxes. We provide robust experimental support for the importance of host activity suggesting that Asian koels actively select occupied boxes.

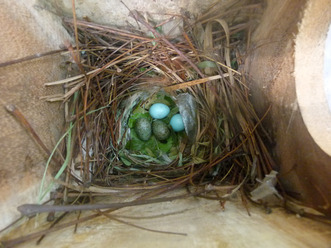

## 1 INTRODUCTION

Obligate avian brood parasites do not build their own nests but lay eggs in the nest of other birds and thereby transfer the costs of rearing its offspring onto another species, the host (Davies, 2000; Moksnes et al., 2000). Brood parasitism substantially reduces reproductive fitness of its host and as a result there is selection for hosts with a better defense (Davies & Brooke, 1989a, 1989b; Rothstein, 1990). Consequently, there should again be strong selection for the parasite to evolve an even better trickery (Davies & Brooke, 1988; Rothstein & Robinson, 1998). In this way, both the brood parasite and its host are involved in a refined evolutionary arms race (Dawkins & Krebs, 1979; Feeney et al., 2014). Globally, avian brood parasites use a large variety of bird species as hosts, for example, the common cuckoo (*Cuculus canorus*) and brown‐headed cowbird (*Molothrus ater*) eggs have been found in the nests of more than 100 different species; however, these two brood parasitic lineages have evolved independently (Campobello & Sealy, 2009; Moksnes & Røskaft, 1995; Ortega, 1998; Rothstein & Robinson, 1998). Moreover, the various nesting species are not equally suitable as hosts. Brood parasites select suitable host species based on their body size, nesting habitat, nest type, nest size, duration of incubation and nestling period, diet, etc. (Hauber et al., 2024; Soler et al., 1998; Stokke et al., 2018). Moreover, the fitness consequences of brood parasites selecting poor hosts can be detrimental to both the parasite and the host species, for example, the brown‐headed cowbird parasitizing nests of American goldfinch (*Spinus tristis*), individuals selecting hosts with inadequate parental care or incompatible feeding habits can lead to reduced reproductive success of the brood parasite (Middleton, 1977, 1991).

However, details on how brood parasites locate and select host nests for parasitism remain poorly understood. Several hypotheses have been suggested to explain the selection of host nests by the brood parasite (Patten et al., 2011).

In our study area, the common myna (*Acridotheres tristis*) is a regular host of the Asian koel (*Eudynamys scolopaceus*), a non‐evictor, generalist brood parasite with general parasitism rates between 31.2% and 33.6% (Begum et al., 2011a, 2011b; Nahid et al., 2021). The common myna usually builds different types of nests in cavities, including holes in trees, inside buildings or roofs, or in the small space between joining palm leaves of coconut (*Cocos nucifera*), fishtail palm (*Caryota urens*), and fountain palm (*Livistona chinensis*) trees. Occasionally common mynas utilize and repair old nests of pied mynas (*Gracupica contra*) or house crows (*Corvus splendens*) (Nahid et al., 2021). However, in our study area, the common myna is not familiar to nest boxes, and hence, nest boxes should constitute unfamiliar but attractive nest sites for common myna. Furthermore, Asian koel is not used to parasitize common myna nests inside nest boxes in our study area. Thus, the common myna‐Asian koel system offers a suitable case for examining the ability of both the host (common myna) and the parasite (Asian koel) to utilize these novel nest sites. We installed 100 wooden nest boxes in our study area to examine whether common mynas and particularly Asian koel would be able to respond to the novel breeding sites. Thus, we aimed to assess to what extent Asian koel were able to parasitize these novel nest sites in our study area. The rate of parasitism would provide data on the ability to locate nests on novel nest sites, which is probably linked to inherited searching behavior in a brood parasite.

## 2 MATERIALS AND METHODS

### 2.1 Study area

Fieldwork for the present experiment was carried out from March 2022 to September 2022 in Jahangirnagar University campus, Bangladesh (23°52′ N, 90°16′ E). In addition, we take advantage of existing data for non‐hosts from this area (during the period 2008–2017, as supportive data). The study area is approximately 280 ha and is a heterogeneous landscape, including woodlands, grasslands, wetlands, cultivated lands, and human settlements (Nahid et al., 2020). Multiple cuckoo species (Asian koel, Indian cuckoo (*Cuculus micropterus*), common hawk cuckoo (*Hierococcyx varius*), jacobin cuckoo (*Clamator jacobinus*), and plaintive cuckoo (*Cacomantis merulinus*)) differing in size from small to large coexist and breed sympatrically in the study area (Nahid, Begum, & Feeroz, 2016; Nahid, Fossøy, et al., 2016).

### 2.2 Study species

The Asian koel is found throughout Asia and is the most common resident among the cuckoo species within this study area (Ali & Ripley, 1981; Begum et al., 2011b; Erritzøe et al., 2012; Nahid, Begum, & Feeroz, 2016; Nahid, Fossøy, et al., 2016; Payne, 2005). Asian koel eggs have been found in 16 passerine species nesting across different habitats and with different nest types (Mann, 2017); however, in our study area, the Asian koel regularly parasitize nests of house crows, common mynas, and long‐tailed shrikes (*Lanius schach*) (Begum et al., 2011a, 2011b; Nahid et al., 2020, 2021). The Asian koel eggs are gray bluish to greenish ground color with brown or black spots and usually lay 2–3 but up to 13 koel eggs have been recorded in one host nest (Erritzøe et al., 2012; Nahid et al., 2021; Payne, 2005).

The common myna is a resident bird of south and Southeast Asia and is one of the most exploited hosts by the Asian koel in our area (BirdLife International, 2017; Craig et al., 2019; also see Table 1). It is an opportunistic omnivorous bird and builds nests close to human habitation as well as natural areas (Ali & Ripley, 1981; Begum et al., 2011b; Lamba, 1963). The breeding season of the common myna lasts from March to August in Bangladesh but can in some areas continue throughout the whole year (Begum et al., 2011a; Nahid et al., 2020). Common myna eggs are immaculate blue, and the clutch size is usually within 4–5 eggs (Nahid et al., 2021). The Oriental magpie robin (*Copsychus saularis*) and jungle myna (*Acridotheres fuscus*) are also common breeding birds within the study area, but there are no records in literature on Oriental magpie robin or jungle myna parasitism by the Asian koel (Erritzøe et al., 2012; Payne, 2005), nor any former records from the study area (Nahid unpublished data).

**TABLE 1:** Summary of the three host species breeding attempts among nest box experiment (*n* = 100 nest boxes) and the number of parasitized and un‐parasitized nests by the Asian koel. Note that none of the non‐host nests were parasitized.

Species	Parasitized nest (%)	Un‐parasitized nest (%)	Total
Common myna	21 (21.2)	78 (78.8)	99
Oriental magpie robin	0	8 (100)	8
Jungle myna	0	6 (100)	6

### 2.3 General methods

**FIGURE 1:**
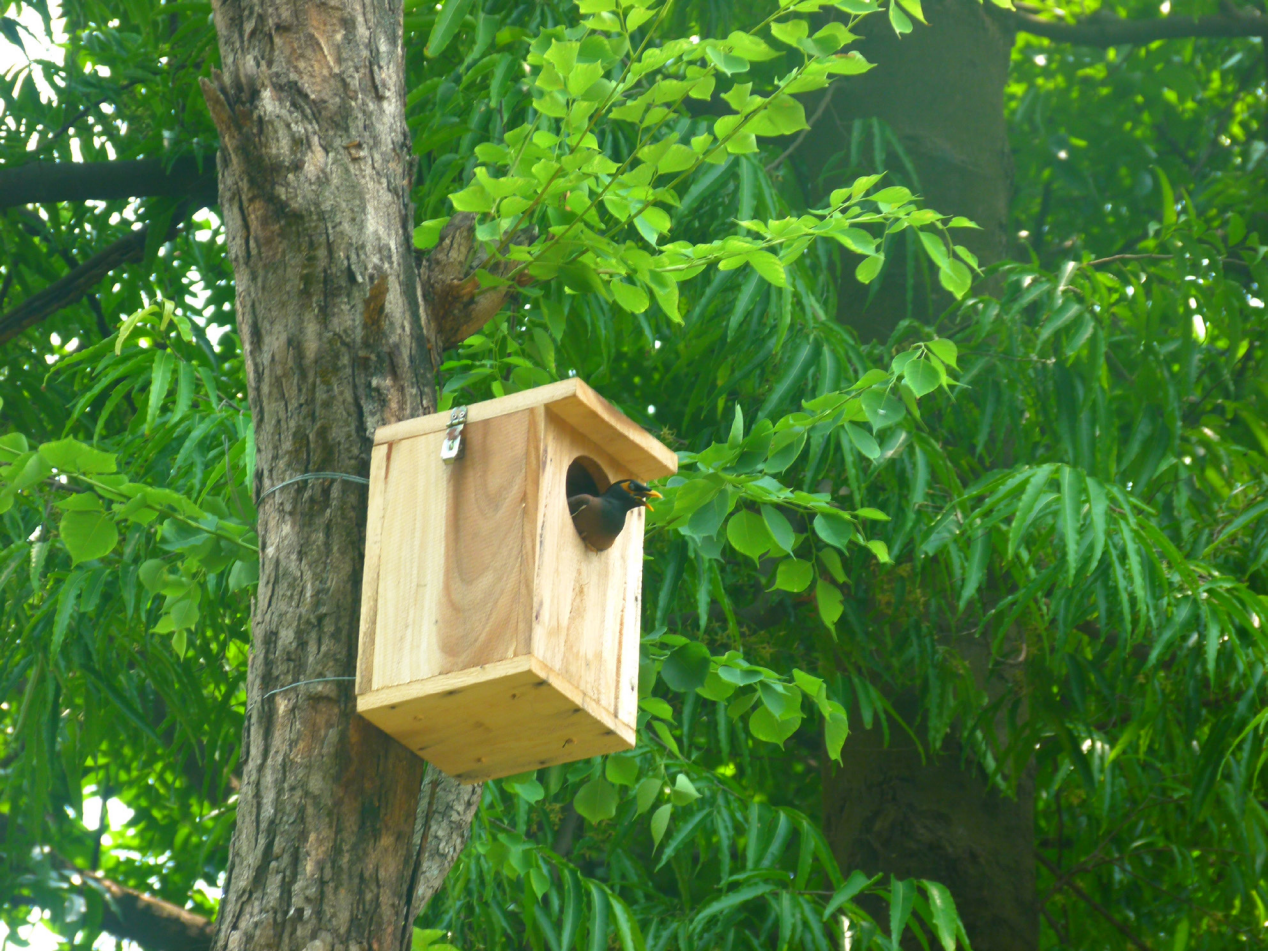
An example of one of the experimental nest boxes, with a common myna in the opening after inspecting the nest box. Experimental nest boxes with two opening diameters; however, the Asian koel was equally effective in parasitizing independent of opening size (see Table S1).

**FIGURE 2:**
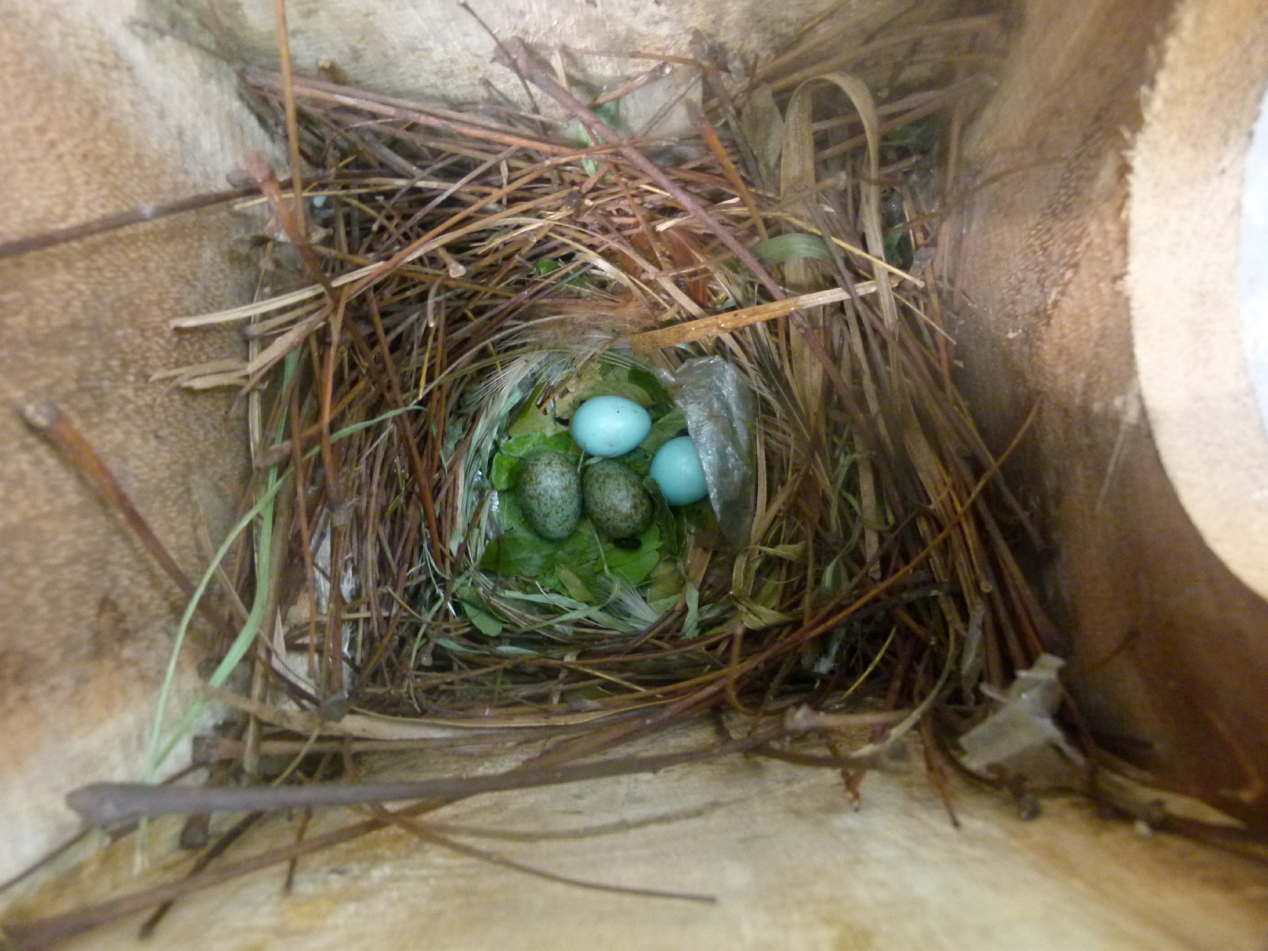
A common myna nest parasitized by the Asian koel. The common myna lays immaculate eggs, resulting in large differences in egg appearance compared with the Asian koel eggs. The Asian koel eggs are regularly accepted despite the large difference in appearance.

We installed 100 nest boxes in the study area in March 2022 (Figure 1). Nest boxes were attached to trees with metal wires about 7 m above the ground. All the nest boxes were placed randomly and located in areas usually utilized by common mynas. The dimensions of the nest boxes were length 20.5 cm, width 20.5 cm, height 30.5 cm, top cover 24.2 cm, and the entrance was 9.5 cm for 50 boxes and 12.7 cm for another 50 boxes. We made the nest entrance in two different sizes as we did not have any data on the minimum opening size that Asian koel would easily be able to enter the nest boxes and successfully parasitize the nest. Importantly, nest box opening had no significant impact on the parasitism rate (Table S1). We monitored the nest boxes regularly (at least once a week) to acquire data on the nest building process by the common myna, and later to examine whether a clutch was parasitized by the Asian koel. Asian koel eggs were easily distinguished from common myna eggs by its color, pattern, and size (Nahid et al., 2021; Figure 2). Moreover, Oriental magpie robin and jungle myna eggs would also easily distinguishable from Asian koel egg (Begum et al., 2012; Karim & Ahsan, 2016; Nahid et al., 2021). Nests that were predated and nests from fledged common myna clutches were regarded as empty nests without parental activity (i.e., inactive favorable nests; *n* = 83). Figures and statistics were performed using R v. 4.3.1 (R Core Team, 2023), with R‐packages “ggstatsplot” (Patil, 2021) and “glmmTMB” (Brooks et al., 2017).

## 3 RESULTS

**FIGURE 3:**
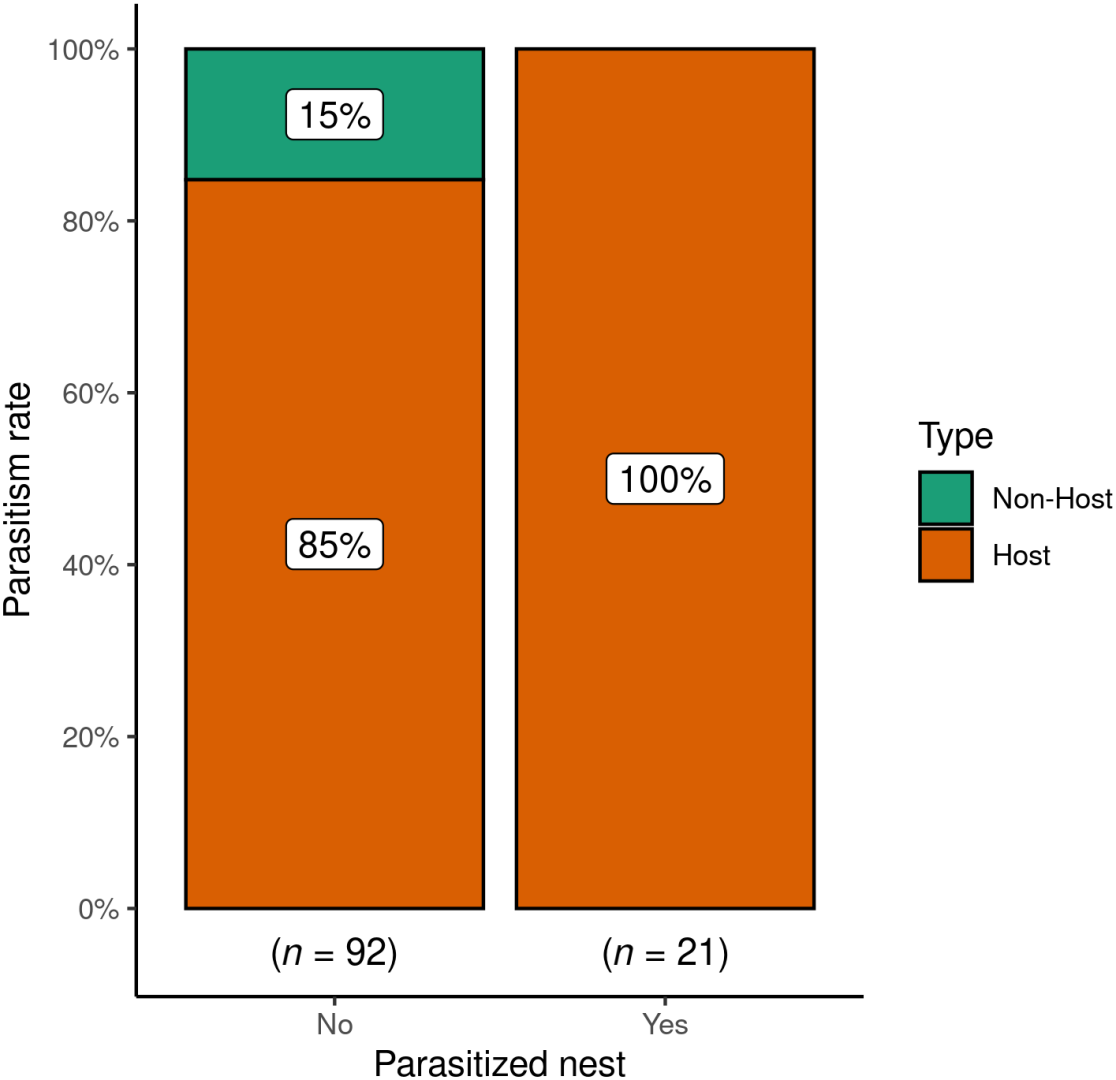
Risk of parasitism by the Asian koel in relation to the nest boxes occupied by host and non‐host species. Orange represents the proportion of host nests and turquoise represents the proportion of non‐host nests. Of 21 parasitized nests, all were common mynas (a regular host), whereas un‐parasitized nests reflect what we expect from host and non‐host species occurrence in the nest boxes. Thus, this figure shows that parasitism occurred exclusively in host nests.

A total of 70 nest boxes out of the 100 installed boxes were occupied at least once during the breeding season. The remaining 30 nest boxes were left empty during the whole study period. A total of 113 nests (common mynas 99 nests across 59 boxes, Oriental magpie robins eight nests across eight boxes and jungle mynas, six nests across six boxes) were found in 70 nest boxes as multiple broods were found (Table 1). Three nest boxes were sequentially used by two different species. During the breeding season, we found 21 nest boxes with two broods, 11 nest boxes with three broods, whereas the remaining 38 nest boxes had just a single brood. A total of 21 (21.2%) common myna nests were parasitized by the Asian koel (Figure 3), whereas none of the Oriental magpie robin and jungle myna nests were parasitized.

We found that parasitism occurred exclusively in active nest boxes, thus Asian koels revealed to not lay eggs randomly in the nest boxes (i.e., independently of host activity around the box). Moreover, we did not find any parasitized nests of non‐hosts; Oriental magpie robins and jungle mynas (Figure 3). Note also that none of an additional 47 Oriental magpie robin‐ nor 29 jungle myna nests, were found parasitized during the period prior to this experiment (2008–2017, Nahid et al. unpublished data), supporting our findings, current literature (Erritzøe et al., 2012; Payne, 2005) and Museum collections (e.g., Tring Museum).

## 4 DISCUSSION

In this study, we examined the ability and extent of adjustment to novel nest sites by hosts and their parasite. We found that the introduced nest sites (i.e., nest boxes; Figure 1) were located and occupied by the common myna and subsequently that a proportion of these were successfully parasitized by the Asian koel. The relatively high proportion of parasitized common myna nests (21.2%) indicate that Asian koels rapidly located host nests even in locally novel nest sites; however, earlier studies show that parasitism rates in this area have proven to be slightly higher for common myna (i.e., 31.2% and 33.6%, respectively; Begum et al., 2011b, Nahid et al., 2021).

None of the empty nest boxes, nor inactive nest boxes containing empty host nests (i.e., successfully fledged or predated nests) were parasitized, indicating that Asian koel closely monitor the potential host and/or nest sites before parasitism, and avoid randomly dumping of eggs into available nests. Thus, our data support that the Asian koel does not select its host solely based on the appearance of the nest. Similarly, the common cuckoo also avoids parasitizing inactive host nests, likely because host activity is an important part of the parasitism process (Yang et al., 2016, 2017; Zhang et al., 2023). Moreover, parasitism of the great spotted cuckoo (*Clamator glandarius*) was as well more regular in active magpie nests during the pre‐laying stage compared with nests without eggs and in the absence of parental activity (Soler & Pérez‐Contreras, 2012).

A closer study of the nest selection process would include tracking individual cuckoo chicks in different environments with GPS transmitters and subsequent monitoring of their host selection behavior when they become mature (Davies, 2015; Yang et al., 2018). Unfortunately, we lack such data and drawing robust conclusions with the present data is however challenging. Thus, with the present data we are unable to rigorously test, for example, the host imprinting hypothesis, the habitat imprinting hypothesis, the natal philopatry hypothesis and any potential impact of imprinting in the present experiment. Nevertheless, our results indicate that the Asian koel closely monitors its host, potentially tracking host activity to successfully locate and time parasitism in available clutches. In the present study, the Asian koel managed to rapidly track its host, even when breeding in locally novel nest sites, achieving approximately regular parasitism rates in its favorable host.

Two other passerines regarded as potential hosts; although not recorded to have been parasitized; Oriental magpie robin and jungle myna also built nests in our nest boxes, but these species remained un‐parasitized suggesting that the Asian koel were able to actively select its favorable host even when breeding in locally novel nest sites. Moreover, existing data on 47 Oriental magpie robin‐ and 29 jungle myna nests with no parasitism events over a 10‐year period prior to this experiment (2008–2017) highlights the discrimination by Asian koel of these two species (see also Erritzøe et al., 2012; Payne, 2005).

In conclusion, we demonstrate nonrandom parasitism by the Asian koel, actively selecting common myna, and only active nests, indicating that the Asian koel selection behavior includes host activity and behavior to effectively locate host nests. Although the underlying mechanism for this host selection remains unknown, the present study contributes to our knowledge of coevolutionary adaptation of an Asian koel‐host system.

## AUTHOR CONTRIBUTIONS

**Mominul Islam Nahid:** Data curation (equal); formal analysis (equal); investigation (lead); resources (equal); writing – original draft (equal). **Peter S. Ranke:** Data curation (equal); formal analysis (equal); methodology (equal); writing – review and editing (equal). **Wei Liang:** Conceptualization (lead); funding acquisition (equal); resources (equal); supervision (equal); validation (equal); writing – review and editing (equal).

## FUNDING INFORMATION

This work was supported by the National Natural Science Foundation of China (Nos 31772453, 31970427 and 32270526 to WL) and by the specific research fund of The Innovation Platform for Academicians of Hainan Province.

## CONFLICT OF INTEREST STATEMENT

The authors declare that they have no competing interests.

## Supporting information


Table S1.

## Data Availability

Data used for this manuscript are available from the Dryad Digital Repository at https://doi.org/10.5061/dryad.8pk0p2ntd.
