# Supplementary material for: Asian koel rapidly locates host breeding in novel nest sites
Source: Ecol Evol. 2024 Apr 29;14(5):e11345. doi: 10.1002/ece3.11345 (PMC11056848; doi:10.1002/ece3.11345)
Supplement: Supplementary file 1 — Table S1. [file ECE3-14-e11345-s001.docx]

**Supplementary material**

**Table S1.** Model output from a generalized binomial mix-effects model, assessing the difference in parasitism probability between small- and medium-sized holes in nest boxes in the study. Estimates showing that hole size did not affect parasitism probability. Response: parasitism (yes/no), fixed-effect: box opening (medium-sized reference level), and nest box identity as random-effect to account for non-independence. Note that there was no effect of box opening size on the probability of being parasitized.

|  | **Estimate** | SE | Z value | *P* |
| --- | --- | --- | --- | --- |
| (Intercept) | -1.99 | 0.40 | -4.93 | <0.001*** |
| Box opening | 0.36 | 0.50 | 0.73 | 0.467 |
